# Term cesarean breech delivery in the first pregnancy is associated with an increased risk for maternal and neonatal morbidity in the subsequent delivery: a national cohort study

**DOI:** 10.1007/s00404-020-05575-6

**Published:** 2020-05-14

**Authors:** Georg Macharey, Anna Toijonen, Pia Hinnenberg, Mika Gissler, Seppo Heinonen, Volker Ziller

**Affiliations:** 1 Department of Obstetrics and Gynecology, University of Helsinki, Helsinki University Hospital, Haartmaninkatu 2, 00029 HUS Helsinki, Finland; 2 National Institute for Health and Welfare (THL), Helsinki, Finland; 3 Department of Obstetrics and Gynecology, University Hospital Marburg, Marburg, Germany

**Keywords:** Breech delivery, Subsequent, Caesarean section, Postpartum hemorrhage, Uterine rupture, Vaginal birth after caesarean

## Abstract

**Purpose:**

To determine whether there is an association between term cesarean breech delivery in the first pregnancy and maternal and neonatal morbidities in the subsequent pregnancy and delivery.

**Methods:**

We conducted a retrospective, nationwide Finnish population-based cohort study, including all deliveries from January 2000 to December 2017. We included all women with the first two consecutive singleton deliveries of which the first one was a breech delivery regardless of mode of delivery (*n* = 11,953), and constructed a data set in which the first two deliveries for these women were connected. The outcomes of the second delivery of the women with a first pregnancy that resulted in cesarean breech delivery at term were compared with women whose first pregnancy resulted in a vaginal breech delivery at term. P-value, odds ratio, and adjusted odds ratio were calculated.

**Results:**

Neonates of a subsequent delivery after cesarean breech delivery had an increased risk for arterial umbilical cord pH below seven, a higher rate of a 5 min APGAR score < 7 and a higher rate of neonatal intensive care unit admission. The women with a history of cesarean section with the fetus in breech presentation were more often in need of a blood transfusion and suffered more often a uterus rupture. In this group, the second delivery was more often a planned cesarean section, an emergency cesarean section, or an instrumental vaginal delivery.

**Conclusions:**

Primary cesarean breech section in the first pregnancy is associated with adverse neonatal and maternal outcomes in the subsequent delivery.

## Introduction

Cesarean section rates are increasing worldwide [1, 2]. The most common reasons for primary cesarean sections are labor arrest, non-reassuring fetal heart rate tracing, and breech presentation (malpresentation) [1, 2]. Breech presentation (malpresentation) in the United States is the most common reason for a planned primary cesarean section [2]. This is most likely due to the association of vaginal breech delivery with an increased risk of short-term neonatal morbidity [3–5]. Many obstetricians and women choose a cesarean section as the mode of delivery to avoid these possible complications, even if the long-term neurological outcome of the infants is normal when mothers and fetuses are well selected, and the deliveries are handled with caution [6, 7]. This decision might seem controversial, as a cesarean section might cause adverse long-term health problems in the offspring, and the uterus scar created by the cesarean section increases the complication risks for the mother during subsequent pregnancies and deliveries [1, 8–12]. Preceding studies indicate that a history of uterine scarring is associated with maternal hemorrhage, placenta accreta, placenta praevia, uterine rupture, stillbirth, and repeated cesarean section in subsequent pregnancies and deliveries [2, 3, 13]. Nonetheless, it seems that women might be unaware of the potential impact of their decision on subsequent deliveries, as the number of planned cesarean sections keeps rising, rather than attempting a cautiously handled trial of vaginal breech delivery for well-selected women as it is possible in Finland. We hypothesized that the subsequent delivery after a primary cesarean section is associated with adverse outcomes, regardless of the mode of it. However, these risks have not been investigated separately for the subsequent delivery after a planned cesarean breech birth compared with a vaginal breech birth. Furthermore, these outcomes are essential to know, and the information should be integrated into the counseling of women with a fetus in breech presentation, especially concerning the following pregnancies and subsequent family planning.

## Methods

### Population

We conducted a retrospective, nationwide Finnish population-based case-control study using data from the Finnish national medical birth register and the hospital discharge register, maintained by the National Institute for Health and Welfare. All Finnish maternity hospitals are contributing clinical data to the register, and reporting to the national registers is obligatory. In Finland, all newborn infants are examined by a pediatrician. Personal identification numbers given at birth can be used to trace the child in the case of death or subsequent hospitalization. The hospital discharge register contains information on procedures and diagnoses (International Statistical Classification of Diseases and Related Health Problems 10th Revision, ICD-10) in the public sector. We included all women undergoing a second term delivery with a history of a singleton term breech delivery during their first delivery regardless of the mode of delivery. We constructed a data set in which the first two deliveries of these women were connected. All women were nulliparous at the time of the first delivery. The first and the second delivery had to be a term delivery. We compared the outcomes of the second delivery of women with a cesarean term breech section during first delivery, with the outcomes of the second delivery of all women with a vaginal term breech delivery during the first delivery. In Finland, pregnant women with one previous lower-segment cesarean section are offered the opportunity to attempt vaginal labor during a subsequent pregnancy. Selecting criteria for a trial of vaginal breech labor in Finland include: fetal size has to be below 4000 g, the fetus is not allowed to have a growth restriction, the maternal pelvis size has to be appropriated, absence of oligohydramnios, the mother is not allowed to have diabetes mellitus type one or two, the fetus has to be in Frank breech or complete breech presentation, the fetal neck has to be flexed. The progress of labor has to be steady, without pathological cardiotocography abnormalities, and the active phase of the second delivery stage is not allowed to extend 60 min. Authorization to use the data was obtained from the National Institute for Health and Welfare as required by the national data protection law in Finland (reference number THL/652/5.05.00/2017).

We compared the labor outcomes of the subsequent delivery of women with a history of breech cesarean section with the labor outcomes of women with a history of vaginal breech delivery. Independent variables were vaginal breech delivery and cesarean breech delivery at the first delivery. The outcomes and variables for the analysis were selected based on previous literature on the subject (Tables 1, 2 and 3). As variables for maternal outcome, we selected maternal mortality, maternal need for blood transfusion, uterus rupture, mode of subsequent delivery planned cesarean, mode of subsequent delivery emergency cesarean section, mode of subsequent delivery instrumental vaginal delivery, mode of subsequent delivery spontaneous vaginal delivery, and mode of subsequent delivery spontaneous vaginal breech delivery (Table 2). As neonatal outcomes, we chose stillbirths during pregnancy, neonatal deaths during delivery, arterial umbilical pH < 7, 5 min APGAR < 4, 5 min APGAR < 7, neonatal intensive unit admission (NICU) admission, neonatal intubation (Table 3).

**Table 1:** Characteristics of the studied women

	Previous cesarean *N* 6414		Previous vaginal *N* 1768		*P*	OR
	*N*	%/SD	*N*	%/SD		
Maternal age < 25	570	9.2	190	10.7	0.017	0.81 (0.68–0.96)
Maternal age ≥ 35	1415	22.8	296	16.7	< 0.001	1.41 (1.23–1.62)
Smoking	693	11.2	183	10.4	0.585	1.05 (0.88–1.25)
Maternal BMI ≥ 30	643	10.3	116	6.6	< 0.001	1.59 (1.29–1.95)
Maternal BMI ≥ 35	214	3.4	36	2.0	0.005	1.66 (1.16–2.37)
Maternal hypothyroidism	31	0.5	9	0.5	0.891	0.95 (0.45–2.00)
Maternal hyperthyroidism	15	0.2	2	0.1	0.324	2.07 (0.47–9.06)
Pregestational insulin-treated diabetes	40	0.6	5	0.3	0.086	2.21 (0.87–5.61)
Gestational diabetes	470	7.6	109	6.2	0.091	1.20 (0.97–1.49)
Preeclampsia/chronic hypertension	207	3.3	25	1.4	< 0.001	2.33 (1.53–3.53)
Placenta praevia	24	0.4	9	0.5	0.428	0.73 (0.34–1.58)
Placenta ablation	23	0.4	4	0.2	0.390	1.59 (0.55–4.60)
PROM	234	3.8	31	1.8	< 0.001	2.12 (1.45–3.10)
Oligohydramnios	91	1.5	14	0.8	0.038	1.80 (1.02–3.17)
Congenital anomalies	325	5.2	90	5.1	0.968	1.00 (0.78–1.26)
Neonatal female gender	3078	49.5	874	49.4	0.281	0.94 (0.85–1.05)
Small for gestational age	76	1.2	24	1.4	0.559	0.87 (0.55–1.38)
Inter delivery interval in months	36.9	21.5	37.8	23.2	0.114	NA
First cesarean was a not planned cesarean section	1887	0	0	0		NA

**Table 2:** Outcome of second delivery of women with a history of planned caesarean section

	Previous cesarean *N* 6414		Previous vaginal *N* 1768		*P*	OR	Adjusted OR
	*N*	%	*N*	%			
Subsequent delivery planned cesarean section^#^	870	13.6	39	2.2	< 0.001	6.96 (5.03–9.63)	6.96 (5.02–9.63)
Subsequent delivery emergency cesarean section^#^	951	14.8	53	3.0	< 0.001	5.63 (4.25–7.47)	6.91 (4.99–9.57)
Subsequent delivery spontaneous vaginal^#^	3792	59.1	1590	89.9	< 0.001	0.16 (0.14–0.19)	0.17 (0.14–0.20)
Subsequent delivery vacuum extraction^#^	794	12.4	34	1.9	< 0.001	7.21 (5.09–10.20)	7.15 (5.05–10.13)
Subsequent delivery vaginal breech delivery^#^	6	0.1	51	2.9	< 0.001	0.03 (0.01–0.07)	0.03 (0.01–0.07)
Maternal blood transfusion*	192	3.0	9	0.5	< 0.001	6.03 (3.08–11.79)	4.95 (2.51–9.79)
Maternal mortality	0	0.0	0	0.0			
Uterine rupture*	137	2.1	7	0.4	< 0.001	5.49 (2.56–11.76)	4.09 (1.88–8.88)

^#^Adjusted for: previous delivery not planned cesarean section, maternal age ≥ 35, maternal BMI ≥ 30, maternal BMI ≥ 35, pregestational diabetes treated with insulin, preeclampsia/chronic hypertension, PPROM, oligohydramnios

*Adjusted for: previous delivery not planned cesarean section, subsequent delivery emergency cesarean section, maternal age ≥ 35, maternal BMI ≥ 30, maternal BMI ≥ 35, pregestational diabetes treated with insulin, preeclampsia/chronic hypertension, PPROM, oligohydramnios

**Table 3:** Outcome of neonates from mothers with previous planned caesarean section

	Previous cesarean *N* 6414		Previous vaginal *N* 1768		*P*	OR	Adjusted OR
	*N*	%	*N*	%			
Neonatal deaths during delivery	6	0.1	5	0.2	0.163	0.50 (0.19–1.35)	
Arterial umbilical pH < 7*	41	0.6	2	0.1	0.001	8.07 (1.95–33.4)	5.66 (1.37–23.46)
5 min APGAR < 4	53	0.8	15	0.4	0.266	1.38 (0.78–2.46)	
5 min APGAR < 7*	172	2.7	29	0.8	< 0.001	2.37 (1.59–3.52)	1.60 (1.08–2.39)
Neonatal NICU admission*	721	11.2	126	3.6	< 0.001	2.47 (2.03–3.01)	1.56 (1.28–1.90)
Neonatal intubation*	55	0.9	10	0.3	0.022	2.16 (1.10–1.31)	1.45 (0.73–2.86)
Stillbirth during pregnancy	16	0.2	6	0.2	0.932	1.04 (0.41–2.67)	

*Adjusted for: previous delivery not planned cesarean section, maternal age ≥ 35, maternal BMI ≥ 30, maternal BMI ≥ 35, pregestational diabetes treated with insulin, preeclampsia/chronic hypertension, PPROM, oligohydramnios, congenital anomalies

We evaluated potential confounders, which could have an effect on the maternal and neonatal outcomes during the second delivery. We reviewed the following factors: maternal age below 25 and over 35 years, smoking, body mass index (BMI) ≥ 30 and ≥ 35, hypo- or hyperthyroidism, gestational diabetes and pre-existing type 1 diabetes mellitus, preeclampsia, placenta praevia, placental abruption, premature rupture of membranes (PROM), oligohydramnios congenital fetal anomalies, infant sex, and small for gestational age (SGA) according to Finnish standards [14, 15].

### Statistical analysis

The calculations were performed using SPSS 19. Statistical differences in categorical variables were evaluated with the Chi-squared test or Fisher’s exact test when appropriate. We calculated odds ratios (ORs) with corresponding 95% confidence intervals using binary logistic regression. A stepwise logistic regression model was done to assess the adjustments. Differences were deemed to be statistically significant with a P-value < 0.05.

## Results

**Fig. 1:**
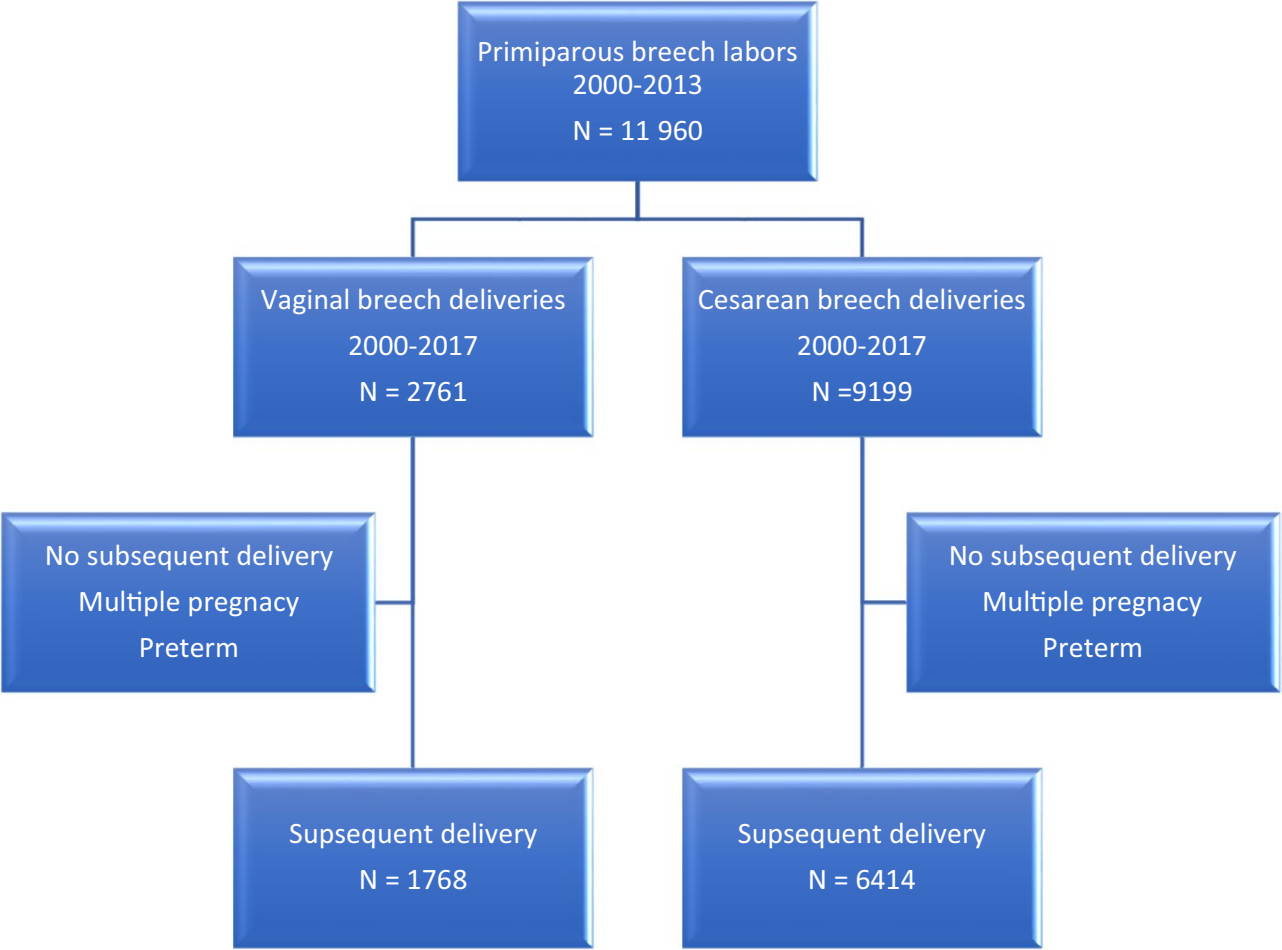
Flow of deliveries through the study period

During the study period, 11,960 women had a second delivery with a history of a singleton term breech delivery as a first delivery. Out of these women, 2761 had a vaginal breech delivery as a first delivery (35%) and 9199 a cesarean Sect. (65%). We were able to match 6414 women who had a subsequent delivery after cesarean breech section and 1768 that were delivered vaginally in the first pregnancy. (Fig. 1).

The maternal characteristics of the studied women are listed in Table 1. Women with a history of cesarean breech delivery were older (maternal age ≥ 35) during the subsequent delivery [odds ratio (OR) 1.41, 95% confidence interval (CI) (1.23–1.62)], they suffered more often from overweight maternal BMI ≥ 30 [OR 1.59, 95% CI (1.29–1.95)] and maternal BMI ≥ 35 [OR 1.66, 95% CI (1.16–2.37)]. These women had more often a higher risk of suffering from preeclampsia/ high blood pressure [OR 2.33, 95% CI (1.53–3.53)] and oligohydramnios [OR 1.80, 95% CI (1.02–3.17)]. A cesarean section during the first delivery was also associated with premature rupture of membranes PROM during subsequent delivery [OR 2.12, 95% CI (1.53–3.53)].

Maternal outcomes are listed in Table 2. Women with a history of cesarean breech delivery had an increased risk of uterus rupture during the subsequent delivery [adjusted odds ratio (aOR) 4.09, 95% CI (1.88–8.88)], or needed more often a blood transfusion [aOR 4.95, 95% CI (2.51–9.79)]. These women had more often a planned cesarean section [aOR 6.96, 95% CI (5.02–9.63)], an emergency cesarean section [aOR 6.91, 95% CI (4.99–9.57)] or an instrumental vaginal delivery (vacuum extraction) [aOR 7.15, 95% CI (5.05–10.13)]. These women were less likely to have a spontaneous vaginal delivery [aOR 0.17, 95% CI (0.14–0.20)], and it was less likely that they would have a vaginal breech delivery [aOR 0.03, 95% CI (0.01–0.07)].

Neonatal outcomes are listed in Table 3. Neonates born by women with a history of cesarean breech delivery had a higher risk of having an umbilical arterial pH below seven [aOR 5.66, 95% CI (1.37–23.46)]. The neonates of mothers with a history of cesarean breech section had a higher rate of 5-min APGAR score < 7 [aOR 1.60, 95% CI (1.08–2.39)] and were more often administrated to the NICU [aOR 1.56, 95% CI (1.28–1.90)].

## Comment

The results of our study show that the total rate of severe maternal and neonatal morbidity is low among women with a history of cesarean breech delivery. Nevertheless, a subsequent delivery after a previous cesarean section with the child in breech presentation is associated with a significantly increased maternal and also neonatal morbidity. Women with a history of cesarean breech birth had a planned cesarean section as mode of delivery for the subsequent birth more often than women with a history of vaginal breech birth. A history of cesarean breech birth was also associated with a seven times higher risk of having an emergency cesarean delivery and a seven times higher risk of needing a vacuum extraction during the subsequent birth. We found that these women had a five-time higher risk of needing a blood transfusion and a nearly four times higher risk of suffering a uterus rupture. The children of them had nearly six times higher risk of having an umbilical arterial pH below seven, compared to the children born after a previous vaginal breech birth. These neonates had a one and a half times higher risk of having a 5 min APGAR score below seven and to be admitted to the NICU.

Our results confirm earlier studies in the fact that a primary cesarean section often leads to a subsequent secondary cesarean section [13]. This might be due to the conviction prevalent among some practitioners “once a cesarean section, always a cesarean section” [16], even if vaginal birth after a cesarean section is safe under certain circumstances [2, 17]. We can also confirm that vaginal birth after a cesarean section is associated with an increased risk of emergency cesarean sections, vacuum extractions, and failure of vaginal delivery, as shown before by a large review of 963 papers from Eden et al. [18]. The vaginal delivery rate after a cesarean section with the fetus in a breech presentation was with 71.5% similar, compared to a general population of vaginal birth after cesarean section for which the successful delivery rate is estimated at 60–80% [18]. A trial of vaginal birth after a cesarean section is usually associated with a 0.5–0.9% risk of uterine rupture [17, 19, 20], our studied women had a four times higher risk of uterine rupture compared to the control group at the rate of 0.4%. The risk of uterine rupture increases; if the inter-delivery interval is less than 12 months, the delivery is post-due-date, the maternal age is over 40 years, the mother suffers from obesity, fetal macrosomia or lower myometrial thickness is decreased (0.6–2.0 mm) [19, 21–26]. Women with a history of cesarean breech section were more often in need of a blood transfusion (five times) regardless of the mode of delivery. This result might be explained by the fact that many of the studied women were undergoing their first trial of vaginal delivery, which is associated with an increased post-partum hemorrhage [20, 27]. The children of women with a vaginal birth after cesarean breech section had a nearly six times higher risk of having an umbilical arterial pH below seven, but overall the risk was low at 0.6%, which is at the same level with the risk of severe neonatal morbidity for nulliparous women in vaginal labor [28, 29]. The neonates also had lower 5 min APGAR points and were more often administrated to the NICU, and this is also most likely due to the fact that a majority of the studied women had their first trial of vaginal labor [28, 29].

Our findings suggest that primipara women undergoing a cesarean breech delivery at term are at increased risk for maternal and neonatal morbidity in the subsequent delivery. Our study adds to the literature on risks associated with a term cesarean section and can assist providers in better counseling of women regarding primary cesarean delivery. Women should be counseled that their decision for a primary cesarean breech delivery is associated with an increased risk of having a subsequent abnormal labor and that their decision increases the risk of uterus rupture during subsequent pregnancy and labor, as it increases the risk of post-partum hemorrhage and adverse neonatal outcome.

The women in our study were primipara when they underwent cesarean breech delivery. It remains uncertain if women that have had a cesarean breech section as second or third delivery, with a history of normal vaginal delivery also suffer from adverse maternal and neonatal outcome during subsequent delivery after cesarean breech delivery. Additionally, it would be interesting to investigate the effect of preterm cesarean breech delivery on subsequent deliveries, as in a preterm cesarean section quite often, a J or U uterotomy is necessary to deliver the child safely.

The strengths of this study include the following: (1) our study is unique since it is the first study, to our knowledge, that reviews adverse outcomes in subsequent labor in women with a history of cesarean breech labor; (2) the analysis is based on a large nationwide population database that allowed us to follow-up successive pregnancies and births to the same woman; (3) access to a linked pregnancy database; (3) large sample size; and (4) robust adjustment for possible confounders.

Our study also has a few limitations that must be considered. The retrospective approach is a limitation of the study; another one is the reliance on data available from the national database. The database has been validated and found to be accurate, but misclassification of data is always possible. However, a misclassification would only have resulted in a minimization of differences in the results.

## Conclusion

Our results show that a subsequent delivery after cesarean breech delivery is associated with an increased maternal and infant morbidity, regardless of the mode of the subsequent delivery. These results must be considered when counseling patients regarding their first breech delivery, as the selected mode of delivery has an effect on subsequent pregnancies and deliveries. Nevertheless, during the counseling of the women, it should become clear that the risk of adverse outcomes is in both groups sporadic. Women with a fetus in breech presentation should be offered external cephalic version and obstetricians need to be trained to offer vaginal breech delivery for well-selected women. It is essential to monitor pregnancies and deliveries among women with a previous cesarean delivery carefully.

## Abbreviations

OR: Odds ratio
aOR: Adjusted odds ratio
CI: Confidence interval
BMI: Body mass index
NICU: Neonatal intensive care unit
PROM: Premature rupture of membranes
SGA: Small for gestational age
